# Salivary Th17 cytokine, human β-defensin 1–3, and salivary scavenger and agglutinin levels in Crohn’s disease

**DOI:** 10.1007/s00784-024-05509-5

**Published:** 2024-01-22

**Authors:** Ulvi Kahraman Gürsoy, Mervi Gürsoy, Vuokko Loimaranta, Jaana Rautava

**Affiliations:** 1 Department of Periodontology, Institute of Dentistry, University of Turku, 20014 Turku, Finland; 2 Welfare Division, Oral Health Care, 20540 Turku, Finland; 3 Department of Oral and Maxillofacial Diseases, Clinicum, Faculty of Medicine, University of Helsinki and Helsinki University Hospital, 00014 Helsinki, Finland; 4 HUS Diagnostic Center, HUSLAB, Helsinki University Hospital, 00260 Helsinki, Finland

**Keywords:** Immunity, Inflammatory bowel diseases, Saliva, T-lymphocytes

## Abstract

**Objectives:**

Crohn’s disease patients, who are prone to develop periodontal diseases, may carry genetic defects in their Th17 cytokine, human beta-defensin (hBD) 1–3, and salivary and scavenger agglutinin (SALSA) expressions. Biochemical composition of saliva reflects the oral consequences of systemic immune response modifications. Our aim was to evaluate the salivary Th17 cytokine, epithelial hBD 1–3, and SALSA levels in relation to Crohn’s disease.

**Materials and methods:**

This cross-sectional study included 42 Crohn’s disease patients and 34 systemically healthy controls. Periodontal and dental indexes were measured, and stimulated saliva samples were collected. Salivary Th17 cytokine levels were analyzed by multiplex technique, and hBD 1–3 and SALSA levels by enzyme-linked immunosorbent assay.

**Results:**

There were 19 gingivitis and 11 initial periodontitis patients in the Crohn’s disease group, and 15 gingivitis and 4 initial periodontitis in the control group. In comparison to controls, higher salivary Th17 cytokine levels were observed in Crohn’s disease patients. No statistical difference was observed between Crohn’s disease and control groups in terms of their salivary hBD 1–3 and SALSA levels. Based on the regression analysis, there is no independent association between Crohn’s disease and salivary Th17 cytokine levels.

**Conclusions:**

Crohn’s disease does not relate to salivary antimicrobial hBD 1–3 or SALSA levels. While Crohn’s disease patients have higher salivary Th17 cytokine levels in comparison to systemically healthy controls, an independent association between Crohn’s disease and Th17 cytokine profile is still missing.

**Clinical relevance:**

Diminished Th17 cytokine response in Crohn’s disease, which might be related to genetic susceptibility, can be also visualized in saliva.

## Introduction

Crohn’s disease is a type of inflammatory bowel disease characterized by the chronic transmural intestinal inflammation. It can affect any part of the gastrointestinal track from the oral cavity to the anus [1]. It has a relapsing–remitting character, and its prevalence varies between 3 and 20 in 100,000 individuals, being highest in the industrialized countries [2]. Major risk factors of the Crohn’s disease are environmental exposures (diet, life style, smoking, and medications), dyscolonization of microbiota, disrupted immune response, and genetic susceptibility. Genetic risk ratio of Crohn’s disease is generally higher than that of other complex diseases, including type 2 diabetes mellitus, schizophrenia, or celiac disease [3]. Single nucleotide polymorphism (SNP)-based genome wide association studies pointed out over 140 loci that are related to the Crohn’s disease [4]. Among the Crohn’s disease-associated genes, a strong and replicated association was observed in *IL23R* gene, which takes part in Th17 lymphocyte differentiation [5]. In turn, high levels of serum interleukin (IL)-6, IL-17A, IL-23, and interferon-γ are commonly observed Crohn’s disease patients, especially when they are at the active state of the disease [6]. Earlier studies also indicated that human beta-defensins (hBDs), which are chemotactic to T cells and induce the mRNA expression of IL-1β, IL-6, and IL-22, may have copy number variations in Crohn’s disease [7]. When tissue and circulating hBD protein levels were analyzed, elevated hBD-3 levels were detected in terminal ileum, and elevated hBD-2 levels were detected in serum samples of Crohn’s disease patients [8]. IL-22 overexpression exacerbates mucosal inflammation and upregulates the expression of the salivary scavenger and agglutinin (SALSA) protein, which is also known as salivary agglutinin SAG, gp340, or DMBT1 [9]. SALSA is an innate immune protein found on mucosal surfaces and in secretions. It interacts with variety of microbial and host ligands, and appears to support immunological homeostasis [9–11]. Indeed, a deletion variant of *DMBT1* with a reduced number of scavenger receptor cysteine-rich domain coding exons is associated with increased risk of Crohn’s disease [12], and diminished SALSA function has been associated with Crohn’s disease [9].

Periodontal diseases and caries are infection-induced diseases of the oral cavity. While dysbiotic biofilms are the prerequisite for the onset of these diseases, systemic and environmental conditions that dysregulate host immune responses contribute to the disease pathogenesis as well [13]. Crohn’s disease patients usually suffer from oral infectious diseases, including apthous-ulcerative lesions, caries, and periodontal diseases [14]. In fact, recent evidence suggests that Crohn’s disease is associated with severe periodontitis and early teeth-loss [15, 16]. On one hand, extension of gut mucosal inflammation was proposed as an explanatory factor for the high prevalence of periodontitis observed in Crohn’s disease [16]. On the other hand, both periodontitis and Crohn’s disease are multifactorial diseases and share similarities in their pathogenesis, including poor oral hygiene, smoking, diet, psychosocial stress, disturbed cytokine network or disrupted antimicrobial response [15, 17]. Indeed, previous studies suggested associations between periodontal inflammation and disrupted intraoral SALSA and hBD levels [18, 19]. Nevertheless, the observed changes on the oral immune response can arouse from the simultaneous impact of Crohn’s disease and oral inflammatory diseases, which is not yet fully elucidated.

Saliva contains large variety of molecules that originate from oral cavity as well as serum-derived markers. Non-invasively collected saliva contains biological markers that are useful to diagnose periodontal diseases and caries, detect oral infection and inflammation in a non-dental setting, and present information about the overall health of the patient [20]. Two common infectious diseases of the oral cavity, periodontitis and caries, can regulate the levels of various salivary biomarkers either by stimulating immune cell responses, or by affecting the oral environment (pH, oxidative stress) or by modulating the salivary microbial composition [21, 22]. Our group recently demonstrated the independent associations between Crohn’s disease and salivary immunoglobulin (Ig) antibody responses against two periodontitis-associated bacteria, *Porphyromonas gingivalis* and *Prevotella intermedia* [23]. In the present study, we hypothesized that the genetic background of Crohn’s disease causes defects in oral immune and antimicrobial protein responses. Thus, we analyzed the salivary Th17 cytokine, epithelial hBD 1–3, and SALSA levels in relation to Crohn’s disease. As the salivary antimicrobial response protein levels can be affected by periodontitis [21] and caries [22], regression models were applied to demonstrate independent associations between Crohn’s disease and salivary Th17 cytokine, epithelial hBD 1–3, and SALSA levels.

## Materials and methods

### Patient recruitment

The patient recruitment, clinical evaluations, and saliva collection of this case–control study were performed between January 2017 and May 2018. The recruitment of Crohn’s disease patients was performed by publishing an invitation advertisement in the patient journal of Crohn and Colitis patient organization (IBD Association of Finland). For all patients, the diagnosis of Crohn’s disease was confirmed by endoscopy and histopathological analysis of intestinal biopsy specimens were prerequisite. To recruit the control group without Crohn’s disease or other immune system disorders, advertisements placed at the Institute of Dentistry, University of Turku.

Exclusion criteria for both groups were as follows: being diagnosed with diabetes mellitus or periodontitis as a manifestation of systemic disease [24], being diagnosed with moderate to severe periodontitis, being smoker, pregnant or lactating, excessive use of alcohol, having less than 24 teeth, and use of antimicrobial medication in the preceding 6 months prior to the clinical examination.

As a result, 42 Crohn’s disease patients and 34 systemically healthy individuals, who were willing to participate and fit to the above mentioned inclusion criteria, were recruited.

### Clinical oral examinations and saliva collection

Oral mucosal examinations of all participants were performed by a specialist in oral pathology (JR). All pathologic findings were registered describing the appearance and location of the lesion. Clinical photographs were taken when necessary.

Fiber-optic transillumination and tactile examination were used during the evaluation of cariological status. Caries lesions, erosion, and attrition were registered. Caries prevalence was described using the decayed-missed-filled teeth (DMFT) index.

A WHO probe (LM-Instruments Oy, Parainen, Finland) with a ball-tip end diameter of 0.5 mm was used in evaluation of periodontal status. Visible plaque index, bleeding on probing, gingival recession, probing pocket depth, and clinical attachment level measurements were performed at six sites per each natural tooth and dental implant. In addition, tooth mobility and furcation defects were recorded when present. The periodontal disease classifications of the patients were determined according to the 2018 Periodontal Disease Classification [25]. All dental and periodontal evaluations were performed by a calibrated periodontist (MG).

### Collection of saliva samples

Participants were asked not to eat or brush their teeth 30 min prior to clinical examinations. Paraffin-stimulated saliva samples were collected from each participant. All appointments were during afternoon hours. Salivary flow rates were recorded. All saliva samples were aliquoted and stored as whole saliva (for cytokine and hBD analysis) or stored with 10 mM EDTA (for SALSA analysis), both at − 70 °C.

### Determination of salivary Th17 cytokine, hBD 1–3, and SALSA levels

Saliva samples were centrifuged at 10,000 rpm for 5 min, and the supernatants were used to detect salivary cytokine and hBD levels. Salivary levels of IL-1β, IL-4, IL-6, IL-10, IL-17A, IL-17F, IL-21, IL-22, IL-23, IL-25, IL-31, IL-33, interferon (IFN)-γ, and soluble CD40L (sCD40L) were measured with the Luminex® xMAP™ technique (Luminex Corporation, Austin, TX, USA) using commercially available kits (Bio-Plex Pro™ Human Th17 Cytokine Panel, Bio-Rad, Santa Rosa, CA, USA). The lowest limit of detection (LOD) for each analyte was as follows: IL-1β, 0.1 pg/ml; IL-4, 1.9 pg/ml; IL-6, 2.0 pg/ml; IL-10, 1.4 pg/ml; IL-17A, 0.8 pg/ml; IL-17F, 3.1 pg/ml; IL-21, 2.5 pg/ml; IL-22, 0.9 pg/ml; IL-23, 5.2 pg/ml; IL-25, 0.8 pg/ml, IL-31, 2.1 pg/ml; IL-33, 1.6 pg/ml; IFN-γ, 0.3 pg/ml; and sCD40L, 4.4 pg/ml.

Salivary hBD-1, hBD-2, and hBD–3 were analyzed using commercial ELISA kits (Peprotech, catalog numbers 900-M202, 900-M172, 900-M210). The analyses were performed according to the manufacturer’s instructions. A 1:30 dilution was used for the hBD-1 analysis, and hBD-2 and hBD-3 were analyzed from undiluted samples. LOD levels for each hBD were as follows: 4 pg/ml for hBD-1, 16 pg/ml for hBD-2, and 62 pg/ml for hBD-3.

Salivary SALSA levels and protein level size variation were analyzed using commercial ELISA kit (Nordic Biosite, Täby, Sweden) and with western blot, respectively. For ELISA assays, the saliva samples were diluted 1:10 or 1:30 in dilution buffer provided with the kit. The analysis was performed according to manufacturer’s instructions. Western blot analysis was done as described earlier [13]. Briefly, saliva samples were separated by SDS–polyacrylamide gel electrophoresis and transferred to a PVDF membrane, 0.2-µm pore size (Bio-Rad). Membranes were blocked with 5% non-fat milk powder in TSB-T (50 mM Tris, 150 mM NaCl, 0.05% Tween 20, pH 7.4) and stained with mAb 213–6 (dil 1:10 000, Bioporto diagnostics, Hellerup, Denmark) and HRP-conjugated anti-mouse antibody (1:10 000, Jackson ImmunoReseach Laboratories, West Grove, PA). The chemiluminescence of the membranes was documented with ChemiDoc MP imaging system (Bio-Rad), and the size of each protein was estimated using Image Lab™ molecular weight analysis tool (version 6.0.1, Bio-Rad Laboratories), and Precisions Plus protein standard (Bio-Rad) transferred to the membrane with the saliva samples.

#### Statistical analyses

All statistical analyses were performed using the SPSS statistical program (version 26.0; IBM Inc., Armonk, NY, USA). The one-way ANOVA test was used for comparing demographic and clinical data (with the exception of gender) between the Crohn’s disease and control groups. Gender distribution between the groups was compared with Fisher’s Exact test. Distribution Th17 cytokines, hBDs, and SALSA levels were skewed; therefore, Mann–Whitney *U* test was applied in data comparisons. Linear regression analyses were performed to analyze the associations between Crohn’s disease and the salivary analytes, after adjusting for age, DMFT, BOP%, and medication use. Statistical significance was defined as *p* < 0.05.

## Results

Nineteen participants were diagnosed with gingivitis, 11 with initial periodontitis, and 12 with periodontal health in the Crohn’s disease group, and 15 participants with gingivitis, 4 with initial periodontitis, and 15 with periodontal health in the control group.

No significant difference was observed between the Crohn’s disease and control groups in terms of their age, gender, number of teeth, VPI%, BOP%, number of teeth with PPD ≥ 4 mm, and saliva flow rates. DMFT index scores, however, were significantly higher in Crohn’s disease group compared to the controls (*p* = 0.001, Table 1).

**Table 1:** Study group characterization based on demographic and clinical (*DMFT*, decayed-missed-filled teeth; *VPI*, visible plaque index; *BOP*, bleeding on probing; *PPD*, probing pocket depth) parameters

	Crohn’s disease *n* = 42	Control *n* = 34	*p*
Age (mean ± st.dev)	46.0 ± 14.1	43.7 ± 10.9	0.434
Gender (male %)	21.4%	26.5%	0.402
No. of teeth (mean ± st. dev)	27.0 ± 2.38	27.9 ± 2.27	0.113
DMFT (mean ± st. dev)	13.8 ± 8.05	7.62 ± 6.71	0.001
VPI % (mean ± st. dev)	23.2 ± 21.7	19.2 ± 16.7	0.380
BOP % (mean ± st. dev)	32.2 ± 24.0	25.2 ± 21.1	0.183
No. of teeth with PPD ≥ 4 mm (mean ± st. dev)	1.26 ± 2.13	0.85 ± 2.4	0.434
Saliva flow ml/min (mean ± st. dev)	1.41 ± 0.75	1.64 ± 0.89	0.218

Elevated salivary IL-4, IL-6, IL-10, IL-17A, IL-17F, IL-22, IL-23, IL-25, IL-35, IFN-γ, and sCD40 levels were observed in Crohn’s disease group, in comparison to controls (Table 2). No difference in salivary levels of IL-1β, IL-31, hBD-1, hBD-2, hBD-3, or SALSA was observed between Crohn’s disease and control groups (Table 3).

**Table 2:** Salivary Th17 cytokine levels of Crohn’s disease and control groups. Data is presented as median (25–75th quartile)

	Crohn’s disease *n* = 42	Control *n* = 34	*p*
IL-1β (pg/ml)	4.71 (1.66–8.16)	3.26 (1.12–6.33)	0.123
IL-4 (pg/ml)	1.97 (1.91–2.16)	1.91 (1.86–1.97)	0.016
IL-6 (pg/ml)	1.41 (0.88–4.47)	0.83 (0.69–1.21)	< 0.001
IL-10 (pg/ml)	1.42 (1.29–1.92)	1.23 (1.17–1.51)	0.001
IL-17A (pg/ml)	0.67 (0.61–0.74)	0.61 (0.56–0.69)	0.011
IL-17F (pg/ml)	2.51 (2.32–2.73)	2.14 (2.14–2.34)	0.02
IL-21 (pg/ml)	9.88 (9.48–10.73)	9.48 (9.10–9.58)	0.088
IL-22 (pg/ml)	3.14 (2.75–3.67)	2.64 (2.37–3.24)	0.004
IL-23 (pg/ml)	1.07 (0.95–1.69)	0.80 (0.64–1.13)	0.002
IL-25 (pg/ml)	0.41 (0.37–0.45)	0.37 (0.36–0.43)	0.018
IL-31 (pg/ml)	29.77 (28.29–34.68)	29.03 (27.41–30.16)	0.112
IL-35 (pg/ml)	6.23 (5.67–6.53)	5.74 (5.61–6.34)	0.035
IFN-γ (pg/ml)	1.72 (1.61–1.90)	1.65 (1.58–1.72)	0.034
sCD40L (pg/ml)	1.0 (0.90–1.34)	0.88 (0.81–1.15)	0.004

**Table 3:** Salivary human beta-defensin (hBD)-1, (hBD-2), (hBD-3), and (salivary scavenger and agglutinin) SALSA levels of Crohn’s disease and control groups. Data is presented as median (25 – 75th quartile)

	Crohn’s disease *n* = 42	Systemically healthy *n* = 34	*p*
hBD-1 (pg/ml)	761 (554–870)	814 (752–935)	0.054
hBD-2 (pg/ml)	489 (296–777)	417 (190–554)	0.056
hBD-3 (pg/ml)	532 (244–737)	548 (482–682)	0.386
SALSA (ng/ml)	7.76 (6.47–10.33)	7.73 (6.18–11.12)	0.942

**Fig. 1:**
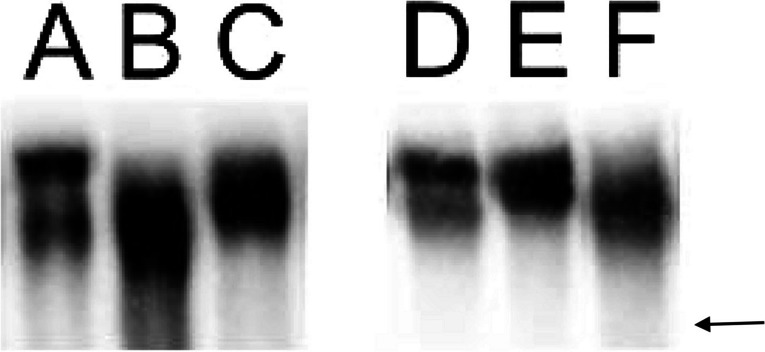
Examples of different size variants of SALSA detected in saliva samples from Chron’s disease patients (**A**–**C**) and controls (**D**–**F**). Western blots of saliva samples were stained with anti-SALSA mAb, and different size of one or two slightly diffuse bands were detected. No difference was noted between the groups. Arrow indicates the 250 kDa standard

In western blots, SALSA appears as slightly diffuse bands, due to its glycosylation. Yet, SALSA proteins with different sizes (270–317 kDa) of one or double bands, were evident in individual saliva samples (examples shown in Fig. 1), but no difference in size patterns was seen between groups.

Linear regression models revealed significant associations between salivary IL-6 and IL-10 with Crohn’s disease; however, those associations were diminished after adjusting the model for age, DMFT, BOP%, and medication use (Table 4). Associations of BOP% and DMFT with salivary Th17 cytokine, hBD 1–3, and SALSA levels are presented in Table 5.

**Table 4:** Unadjusted and adjusted (age, DMFT, BOP%, use of medications) associations of salivary Th17 cytokines with Crohn’s disease. Data are presented as unstandardized B, standardized *β* coefficient, 95% confidence interval for *B*, and *p* values

	Unadjusted	Adjusted
IL-4	*B*, 0.172; *β*,0.208; 95% CI, − 0.016–0.359; *p*, 0.072	*B*, 0.069; *β*, 0.084; 95% CI, − 0.240–0.379; *p*, 0.656
IL-6	*B*, 2.243; *β*, 0.287; 95% CI, 0.506–3.980; *p*, 0.012	*B*, 1.301; *β*, 1.66; 95% CI, − 1.529–4.130; *p*, 0.362
IL-10	*B*, 0.291; *β*, 0.237; 95% CI, 0.015–0.567; *p*, 0.039	*B*, 0.074; *β*, 0.060; 95% CI, − 0.381–0.528; *p*, 0.747
IL-17A	*B*, 0.061; *β*, 0.181; 95% CI, − 0.016–0.138; *p*, 0.118	*B*, − 0.023; *β*, − 0.070; 95% CI, − 0.148–0.101; *p*, 0.708
IL-17F	*B*, 0.607; *β*, 0.198; 95% CI, − 0.088–1.303; *p*, 0.086	*B*, 0.215; *β*,0.070; 95% CI, − 0.928–1.359; *p*, 0.708
IL-22	*B*, 0.365; *β*, 0.083; 95% CI, − 0.645–1.374; *p*, 0.474	*B*, − 0.079; *β*, − 0.018; 95% CI, − 1.752–1.594;* p*, 0.925
IL-23	*B*, 0.462; *β*, 0.122; 95% CI, − 0.405–1.329, *p*, 0.292	*B*, − 0.344; *β*, − 0.091; 95% CI, − 1.765–1.077; *p*, 0.631
IL-25	*B*, 0.054; *β*, 0.182; 95% CI, − 0.014–0.122; *p,* 0.116	*B*, 0.009; *β*, 0.031; 95% CI, − 0.103–0.121; *p*, 0.869
IL-31	*B*, 4.893; *β*, 0.167; 95% CI, − 1.797–11.584; *p*, 0.149	*B*, 2.489; *β*, 0.085; 95% CI, − 8.564–13.543; *p*, 0.655
IL-35	*B*, 0.535; *β*, 0.145; 95% CI, − 0.308–1.378; *p*, 0.210	*B*, 0.096; *β*, 0.026; 95% CI, − 1.298–1.490; *p*, 0.891
IFN-γ	*B*, 0.255; *β*, 0.204; 95% CI, − 0.028–0.539; *p*, 0.076	*B*, − 0.433; *β*, − 0.177; 95% CI, − 1.363–0.498; *p*, 0.357
sCD40L	*B*, − 0.105; *β*, − 0.043; 95% CI, − 0.672–0.462; *p*, 0.713	*B*, − 0.046; *β*, − 0.019; 95% CI, − 0.702–0.611; *p*, 0.890

**Table 5:** Associations of BOP% and DMFT with salivary Th17 cytokine, hBD 1–3, and SALSA levels. Data are presented as unstandardized *B*, standardized* β* coefficient, 95% confidence interval for *B*, and *p* values (*p* values < 0.05 are marked with bold font)

	Crohn’s disease		Controls	
	BOP%	DMFT	BOP%	DMFT
IL-1β	*B*, − 5.33; *β*, − 1.19; 95% CI, − 11.8–1.10; *p*, 0.097	*B*, − 0.984; *β*, − 0.721; 95% CI, − 2.34 – 60.9, *p*, 0.140	*B*, 1.59; *β*, 0.319; 95% CI, − 7.39 – 10.6; *p*, 0.697	*B*, 0.280; *β*, 0.177; 95% CI, − 1.31 – 1.87; *p*, 0.700
IL-4	*B*, − 30.23; *β*, − 0.68; 95% CI, − 272–212; *p*, 0.792	*B*, − 15.9; *β*, − .16; 95% CI, − 66.8 – 35.1; *p*, 0.512	*B*, 168; *β*, 1.62; 95% CI, − 170 – 508; *p*, 0.290	*B*, − 3.71; *β*, − 0.113; 95% CI, − 63.7 – 56.3; *p*, 0.892
IL-6	*B*, 3.07; *β*, 0.267; 95% CI, − 8.46–14.6; *p*, 0.575	*B*, − 1.504; *β*, − 0.429; 95% CI, − 3.927 – 0.920; *p*, 0.203	*B*, − 29.8; *β*, − 1.99; 95% CI, − 101 – 42.1; *p*, 0.372	*B*, 8.82; *β*, 1.87; 95% CI, − 3.89 – 21.5; *p*, 0.151
IL-10	*B*, 29.9; *β*, 0.977; 95% CI, − 47.5–107; *p*, 0.419	*B*, 13.3; *β*, 1.42, 95% CI, − 2.99 – 29.5; *p*, 0.102	*B*, 45.7; *β*, 0.829; 95% CI, − 127 – 219; *p*, 0.566	*B*, − 16.4; *β*, − 0.938; 95% CI, − 47.0 – 14.3; *p*, 0.258
IL-17A	*B*, − 105; *β*, − 0.890; 95% CI, − 381–169; *p*, 0.421	*B*, − 27.5; *β*, − 0.756; 95% CI, − 85.4 – 30.4; *p*, 0.323	*B*, − 4.15; *β*, − 0.025; 95% CI, − 708 – 699; *p*, 0.990	*B*, − 68.0; *β*, − 1.29; 95% CI, − 192 – 56.5; *p*, 0.248
IL-17F	*B*, − 5.21; *β*, − 0.459; 95% CI, − .6–59.2; *p*, 0.864	*B*, − 5.14; *β*, − 1.48; 95% CI, − 18.7 – 8.4; *p*, 0.427	*B*, 27.8; *β*, 0.459; 95% CI, − 71.1 – 126; *p*, 0.540	*B*, 1.54; *β*, 0.080; 95% CI, − 15.9 – 19.0; *p*, 0.846
IL-21	*B*, − 5.047; *β*, − 0.792; 95% CI, − 33.2 – 23.07; *p*, 0.704	*B*, 0.753; *β*, 0.386; 95% CI, − 5.16 – 6.66; *p*, 0.788	*B*, − 3.21; *β*, − 0.166; 95% CI, − 99.5 – 93.1; *p*, 0.942	*B*, − 16.8; *β*, − 2.75; 95% CI, − 33.8 – 0.215; *p*, 0052
IL-22	*B*, 2.204; *β*, 0.217; 95% CI, − 41.9 – 46.3; *p*, 0.916	*B*, 6.04; *β*, 1.94; 95% CI, − 3.24 – 15.3; *p*, 0.183	*B*, − 17.5; *β*, − 0.575; 95% CI, − 101 – 66.1; *p*, 0.648	*B*, 17.9; *β*, 1.87; 95% CI, 3.16 – 32.7; ***p***, **0.023**
IL-23	*B*, − 19.9, *β*, − 1.340, 95% CI, − 85.3 – 45.3, *p*, 0.520	*B*, − 8.62; *β*, − 1.89; 95% CI, − 22.4 – 5.12; *p*, 0.198	*B*, 16.32; *β*, 0.888; 95% CI, − 35.6 – 68.3; *p*, 0.495	*B*, − 17.4; *β*, − 2.99; 95% CI, − 26.6 – 8.23; ***p***, **0.002**
IL-25	*B*, − 86.6; *β*, − 0.737; 95% CI, − 847 – 674; *p*, 0.810	*B*, 6.71; *β*, 0.186; 95% CI, − 153 – 166; *p*, 0.929	*B*, − 104; *β*, − 0.260; 95% CI, − 1411 – 1203; *p*, 0.861	*B*:161; *β*:1.27; 95% CI, − 69.7 – 392; *p*, 0.148
IL-31	*B*, 0.569; *β*, 0.480; 95% CI, − 7.53 – 8.67; *p*, 0.882	*B*, − 0.967; *β*, − 2.67; 95% CI, − 2.67 – 0.736; *p*, 0.242	*B*, − 4.51; *β*, − 1.18; 95% CI, − 15.6 – 6.54; *p*, 0.380	*B*, − 1.18; *β*, − 0.99; 95% CI, − 3.14 – 0.773; *p*, 0.204
IL-35	*B*, 10.56; *β*, 1.044; 95% CI, − 28.5 – 49.6; *p*, 0.569	*B*, 0.707; *β*, 0.228; 95% CI, − 7.49 – 8.91; *p*, 0.855	*B*, − 42.5; *β*, − 1.48; 95% CI, − 125 – 40.7; *p*, 0.278	*B*, 16.5; *β*, 1.81; 95% CI, 1.76–31.2; ***p***, **0.032**
IFN-γ	*B*, 52.6; *β*, 1.921; 95% CI, − 92.9 – 198; *p*, 0.449	*B*, 30.4; *β*, 3.62; 95% CI, − 0.244 – 60.9; *p*, 0.052	*B*, 37.5; *β*, 1.467; 95% CI, − 96.4 – 171; *p*, 0.542	*B*, 37.6; *β*, 1.05; 95% CI, 1.37 – 73.9; ***p***, **0.043**
sCD40L	*B*, 85.5; *β*, 0.880; 95% CI, − 23.7 – 194; *p*, 0.115	*B*, 17.5; *β*, 0.588; 95% CI, − 5.47 – 40.4; *p*, 0.124	*B*, 37.5; *β*, 1.47; 95% CI, − 96.4 – 171; *p*, 0.542	*B*, 3.71; *β*, 0.458; 95% CI, − 19.9 – 27.4; *p*, 0.731
hBD-1	*B*, − 0.28; *β*, − 187; 95% CI, − 0.114 – 0.058; *p*, 0.490	*B*, 0.015; *β*, 0.316; 95% CI, − 0.003 – 0.033, *p*, 0.104	*B*, − 0.028; *β*, − 0.338; 95% CI, − 0.123 – 0.066; *p*, 0.517	*B*, 0.01;* β*, 0.393; 95% CI, − 0.006 – 0.027; *p*, 0.194
hBD-2	*B*, 0.054; *β*, 0.937; 95% CI, 0.009 – 0.099; ***p***,** 0.022**	*B*, 0.007; *β*, 0.382; 95% CI, − 0.003 – 0.016; *p*, 0.146	*B*, 0.005; *β*, 0.093; 95% CI, − 0.055 – 0.065; *p*, 0.848	*B*, 0.004; *β*, 0.251; 95% CI, − 0.006 – 0.015; *p*, 0.364
hBD-3	*B*, − 0.002; *β*, − 0.029; 95% CI, − 0.046 – 0.042; *p*, 0.918	*B*, 0.002; *β*, 0.100; 95% CI, − 0.007 – 0.011; *p*, 0.611	*B*, 0.026; *β*, 0.160; 95% CI, − 0.136 – 0.187; *p*, 0.729	*B*, 0.023; *β*, 0.450; 95% CI, − 0.006 – 0.051; *p*, 0.106
SALSA	*B*, − 2.31; *β*, − 0.370; 95% CI, − 7.71 – 3.08; *p*, 0.371	*B*, 0.316; *β*, 0.165; 95% CI, − 0.818 – 1.45; *p*, 0.558	*B*, 0.345; *β*, 0.060; 95% CI, − 7.21 – 7.90; *p*, 0.920	*B*, − 0.470; *β*, − 0.259; 95% CI, − 1.81 – 0.865; *p*, 0.446

## Discussion

To our knowledge, this study is the first to analyze salivary hBD-1, hBD-2, hBD-3, and SALSA levels in Crohn’s disease patients. According to our results, salivary hBD-1, hBD-2, hBD-3, and SALSA levels of Crohn’s disease patients are similar to those of systemically healthy controls, while salivary Th17 cytokine levels were found higher in Crohn’s disease patients. Nevertheless, according to the regression analysis, there is no independent association between elevated salivary Th17 cytokine levels and Crohn’s disease.

Comprehensive analysis of salivary Th17 cytokine, hBDs, and SALSA levels were the main strength of this study. Previous studies indicated that there are reciprocal interactions between the expressions of Th17 cytokine, hBD 1–3, and SALSA levels [7–9]. One limitation of this study was the relatively low participant numbers. Participants with Crohn’s disease were recruited from the Crohn and Colitis patient organization (IBD Association of Finland) between January 2017 and May 2018. All members of the association received the invitation and all who fit to the inclusion criteria were recruited. Therefore, no sample size calculation was performed. Secondly, the localization of the lesions and disease behavior according to the Montreal classification were not available; therefore, this information was not implemented into the current study. Thirdly, even though the periodontal disease diagnosis of participants was determined according to the 2018 Periodontal Disease Classification [25], use of WHO probe and the lack of radiographs restrained us to define the grades of periodontitis. As the maximum probing pocket depth measured from each periodontitis patient was 4 mm, these participants were diagnosed as initial periodontitis. Finally, majority of the Crohn’s disease patients were receiving anti-inflammatory or immunosuppressive drugs. While this was a standard procedure for this group of patients, it may still affect the cytokine and antimicrobial peptide expression profiles.

Th17 cytokine levels were previously evaluated in saliva, gingival crevicular fluid, dental biofilm, and gingival tissue samples of Crohn’s disease patients. Elevated gingival IL-10 RNA expression was documented in Crohn’s disease patients in relation to healthy controls, while no difference was observed for IL-2 and IL-6 [26]. When the gingival crevicular fluid Th17 cytokine protein expressions were analyzed in Crohn’s patients, similar expression profiles of IL-1β, IL-6, IL-10, IL-12p40, IL-12p70, IFN-γ, and tumor necrosis factor (TNF)-α were observed in Crohn’s disease patients and in systemically healthy controls [27]. According to our findings, salivary Th17 cytokine levels were elevated in Crohn’s disease patients; however, there are no independent associations between Crohn’s disease and elevated cytokine levels. Although a previous study showed higher salivary IL-1β and TNF-α levels in Crohn’s patients in comparison to systemically healthy controls, no information on the oral health status of the participants was given [28]. Indeed, similar to our findings, when the salivary samples were collected from individuals with healthy periodontium, no significant difference was observed in IL-1β, IL-10, IL-17A, and TNF-α levels between Crohn’s patients and healthy controls [29]. In inflammatory bowel disease patients, gingival and intestinal tissue Th17 cytokine expression profiles differ from each other [30], which indicates that the systemic condition is not the only determinant of oral cytokine expression, but the local environment, i.e., oral microbiome, also plays a significant role.

As mentioned above, to our knowledge, there is no available information on the oral hBD or SALSA levels of Crohn’s disease patients. hBDs are antimicrobial peptides with wide-range of functions, including the regulation of T cell chemotaxis, epithelial cell proliferation, and wound healing. In the oral cavity, hBDs are mainly secreted by epithelial cells [31]. Early studies associated Crohn’s disease with low copy number defects at the hBD-2 gene (*DEFB4*) region [7, 32]. Interestingly, studies by Fellermann et al., [7] and Bentley et al., [32] found the associations in opposite directions. However, neither genome wide association studies nor replication studies found evidence on the association between *DEFB4* gene copy number variation and Crohn’s disease, indicating the false-positive results were possibly an outcome of limited statistical power and technological limitations [32, 33]. Similarly, an earlier study proposed association between Crohn’s disease and copy number defect at the gene *DMBT1* [12] but this was not confirmed in a later study with other cohort [34]. In our study, we could see size variations of the salivary SALSA molecules between individuals, but no difference was observed between Crohn’s disease patients and control group. The noted size variation of SALSA may reflect to differences in protein core, but also the heavy glycosylation of SALSA affects the protein size and thus hampers the interpretation. Increased SALSA levels are reported in the intestine of Crohn’s disease patients [12], but based on our findings, salivary hBD-1, hBD-2, hBD-3, or SALSA levels did not differ between Crohn’s disease patients and systemically healthy controls. This is in line with the recent evidence that defects in the hBD or SALSA expressions do not necessarily exists in Crohn’s disease, as there is no genetic susceptibility. Yet, confounding factors, such as disease-specific quality of life, may significantly contribute to the dual interactions between Crohn’s disease and periodontal immune responses [35].

## Conclusion

Our findings suggest that there is a tendency of increased salivary Th17 cytokine levels in Crohn’s disease patients; however, an independent association between Crohn’s disease and Th17 cytokine profile is lacking. The shifts in the oral cytokine levels of Crohn’s disease patients can be either an outcome of genetic background, or explained by the existence of periodontal diseases and mucosal lesions that are related to Crohn’s disease, or both.
